# Expression Patterns of CREBs in Oocyte Growth and Maturation of Fish

**DOI:** 10.1371/journal.pone.0145182

**Published:** 2015-12-23

**Authors:** Balasubramanian Senthilkumaran, Gunti Sreenivasulu, De-Shou Wang, Cheni-Chery Sudhakumari, Tohru Kobayashi, Yoshitaka Nagahama

**Affiliations:** 1 Department of Animal Biology, School of Life Sciences, University of Hyderabad, P.O. Central University, Hyderabad, 500 046, Telangana, India; 2 Laboratory of Reproductive Biology, National Institute for Basic Biology, Myodaiji, Okazaki, 444–8585, Japan; Shanghai Ocean University, CHINA

## Abstract

In fish, oocyte meiotic maturation is regulated by 17α, 20β-dihydroxy-progesterone through cAMP. To study the role of cAMP response element binding protein (CREB) in meiotic maturation, we cloned and characterized the expression pattern of CREBs from two fish models, the Nile tilapia and catfish. In the Nile tilapia three different CREBs were identified where in CREB1 was found in many tissues including gonads with abundant expression in testis. CREB2, few amino acids shorter than CREB1, was expressed in several tissues with abundant expression in ovary. In addition, a 3’UTR variant form, CREB3 was exclusively found in ovary. During natural 14-day ovarian cycle of the Nile tilapia, CREB1 expression was stable throughout vitellogenesis with a sharp decrease on the day of spawning. In contrast, CREB2 remain unchanged throughout the ovarian cycle, however elevated in 11-day full-grown immature ovarian follicle and after hCG-induction. Interestingly, CREB3 expression was induced three folds on the day of spawning as well as during hCG-induced oocyte maturation. Based on the synergistic expression pattern, CREB1 is likely to control oocyte growth, whereas CREB 2 and 3 contribute to oocyte maturation in tilapia and the latter seems to be critical. In catfish, a single form of CREB showed a maximum expression during spawning phase and hCG-induced maturation both *in vivo* and *in vitro* augmented CREB expression. These results suggest that spatial and temporal expression of CREBs seems to be important for final oocyte maturation and may also regulate oocyte growth in fish.

## Introduction

Oogenesis in teleosts in its broadest sense comprises of two phases, oocyte growth (vitellogenesis) and final maturation (resumption of meiosis) that is regulated by follicle stimulating hormone (FSH) and luteinizing hormone (LH) respectively [1]. In certain cases, LH regulates both the processes with very less role for FSH [2,3]. It is well established that, estradiol 17β (E_2_) produced in ovarian granulosa cells by the enzyme ovarian cytochrome P450 aromatase (*Cyp19a1a*), largely controls vitellogenesis [1]. On the other hand, oocyte maturation is promoted [1] by the maturation inducing steroid, 17α, 20β-dihydroxyprogesterone (17α, 20β-DP), produced in ovarian granulosa cells by 20β-hydroxysteroid dehydrogenase (*20β-HSD*). Plasma E_2_ levels rise gradually throughout oocyte growth and decreases suddenly with the onset of oocyte maturation while 17α, 20β-DP levels stays basal during oocyte growth and increases sharply with maturation [4]. This shift in steroidogenesis seems to be a critical step during oocyte maturation in several teleost species [1,5].

Molecular mechanisms governing shift in steroidogenesis were studied in greater details in tilapia and to some extent in catfish model [1,5,6]. In tilapia, consistent with plasma steroid levels, a gradual increase in expression and activity of *Cyp19a1a* throughout vitellogenesis and a diminished or undetectable activity and expression in post-vitellogenic ovarian follicles/during meiotic maturation was found [7]. A down-regulation of *Cyp19a1a* expression was noticed when post-vitellogenic follicles of tilapia were incubated with hCG, *in vitro* [7]. Further studies demonstrated that AD4BP/SF-1 and FOXL-2 regulate the expression *Cyp19a1a* [7–9]. An increase in *20β-HSD* mRNA and/or activity is known in ayu, catfish and trout [10–12] during oocyte maturation, whereas in tilapia, *20β-HSD* expression was found to be basal in vitellogenic follicles, undetectable in post-vitellogenic follicles and a rise in expression was observed during oocyte maturation [13]. Based on these studies, it is suggested that shift in steroidogenesis is governed by subjugation of *Cyp19a1a* expression and an increase in *20β-HSD* expression [1,5].

Transcriptional regulation of *Cyp19a1a* is relatively well studied [7,8,14]. However, *20β-HSD* promoter was not analyzed explicitly except for a study from our laboratory [15]. We have previously reported the characterization of *Cyp19a1a* promoter and its regulation by Ad4BP/SF-1 [7] and *20β-HSD* promoter by cAMP responsive elements [15]. Since, expression of both *20β-HSD* and *Cyp19a1a* in granulosa cells is modulated by gonadotropins via cAMP, understanding how cAMP controls the up-regulation of *20β-HSD* and down regulation of *Cyp19a1a* during steroidogenic shift is interesting. cAMP responsive elements have been identified on both *20β-HSD* [15] and *Cyp19a1a* promoter motifs [7,14], hence transcriptional regulation of these genes during shift in steroidogenesis could possibly occur by spatial and temporal expression of CREBs. Present study is intended to test the possibilities of how CREB’s regulate the shift in steroidogenesis vis-à-vis final oocyte maturation by characterizing the expression pattern. We have identified multiple forms of CREBs from gonads of Nile tilapia (*Oreochromis niloticus*) and a single form of CREB from air-breathing catfish (*Clarias gariepinus*) and characterized their expression patterns during natural and gonadotropin (hCG) induced final oocyte maturation. To our knowledge, this is the first report to implicate a pivotal role for CREBs in meiotic maturation in any lower vertebrates.

## Materials and Methods

### Animals

The Nile tilapia has a fortnight spawning cycle and is an excellent model to study oocyte growth and maturation events. The average ovarian cycle is of 14–18 days within which the ovarian follicles undergo different developmental stages. Ovarian follicle begins early vitellogenesis (1–4 days) a day after ovulation and passes through mid vitellogenic stage at about 5–7 days and becomes full grown immature follicle by 8 – 11^th^ day. Spawning usually occurs on 14^th^ day and in some cases it may extend up to 18^th^ day [7,13]. The Nile tilapias were reared and maintained as described earlier and ovarian follicles at different stages of natural ovarian cycle were collected after careful observation and utilized for gene expression analysis [13]. Experiments involving hCG-induced oocyte maturation are done as described earlier using 11 day ovarian follicles isolated from tilapia which has 14-day spawning cycle tested at least for three consecutive durations as explained earlier [13].

Air-breathing Catfish, *Clarias gariepinus* has an annual ovarian cycle. Preparatory phase (January–April) in which vitellogenesis begins and passes through mid vitellogenic stage. In pre-spawning phase (May–June) ovarian follicles develop through late vitellogenic stage and become full-grown immature ovarian follicles ready to be spawned. The spawning phase lasts typically in July–October that matches with Monsoon season in the South India and we observed an extended spawning period that overlaps with post-spawning phase of catfish in the North Indian region. Ovary in the months of November–December is generally in resting phase. Catfish used in this study were purchased locally from farmers. They were maintained under normal photoperiod and ambient temperature conditions in aquarium tanks during acclimation and experimentation. Feeding as well as rearing of catfish and the hCG-induced oocyte maturation experiments are performed as described earlier [13]. All the experiments conducted on the Nile tilapia and catfish were following general guidelines of Institutional Animal Ethical Committee (IAEC, National Institute of Basic Biology, Japan and University of Hyderabad, India respectively). An approval was not required for the edible fish sacrifice.

### Cloning and sequence analysis of CREB cDNAs

#### RT-PCR amplification of partial cDNAs homologous to CREB

Based on nucleotide sequences of vertebrate CREB’s, sets of degenerate primers were (Table 1) designed and used in RT-PCR amplification of ovarian first-strand cDNA preparation. The amplicons were cloned in pGEM-T easy (Promega) vector and subsequently sequenced. The similarity of cloned sequences was assessed by performing BLAST analysis.

**Table 1:** Oligonucleotide sequences used in cloning and expression analysis of tilapia and catfish CREBs.

Primer	Sequence 5' to 3'	Purpose
DF1	CATMTATCAGACYAGCASSGGSCA	For amplification of partial CREB cDNA from Tilapia and catfish.
DR1	CYTTCTTCTTCCTGCGACACTC	For amplification of partial CREB cDNA from Tilapia and catfish.
NT CREB1-F	GGCACAGATTGCTACTTTGG	For RT-PCR of Tilapia CREB 1
NT CREB1-R	CAGGTGTGGCAGCAGCAGC	For RT-PCR of Tilapia CREB 1
NT CREB2-F	GGAGTACGTGAAGTGTCTGGAG	For RT-PCR of Tilapia CREB 2
NT CREB2-R	CTGATGGTTGATTTCAAATTGCTC	For RT-PCR of Tilapia CREB 2
NT CREB3-F	CTGGGTAAATCTACCGCTCATC	For RT-PCR of Tilapia CREB 3
NT CREB3-R	GAACATTTGTTTGTTTTAATATATG	For RT-PCR of Tilapia CREB 3
CF GSP-R1	AGTTTGCAGCCCTTGCACGCCGTC	For 5' RACE of catfish CREB
CF GSP-R2	CTGGATGGCTCCACCCTGTGTGAT	For 5' RACE of catfish CREB
CF GSP-F1	CGCCTCATGAAGAACAGGGAAGC	For 3' RACE of catfish CREB
CF GSP-F2	AGGGAAGCGGCCCGAGAGTGTCGC	For 3' RACE of catfish CREB
CF qRT-F	CGTCCTTCTTACAGGAAGATCC	For Real-time RT-PCR of catfish CREB
CF qRT-R	TCTCTGAGCTGTATTTGGCACG	For Real-time RT-PCR of catfish CREB

#### cDNA library construction and screening

cDNA libraries from tilapia ovary and testis were constructed as described earlier [13]. In brief, 5 μg of poly (A)^+^ RNA was isolated from ovarian follicles and a λl-ZAP library was constructed. The library was packaged into UNI-ZAP XR using Gigapack II gold packaging extract as per the manufacturer instructions. Partial cDNAs cloned (described in the above section) were labeled with α-P^32^ using random hexa-nucleotide kit. Libraries were screened with the probe under high stringency conditions for three rounds. After three rounds of screening, positive clones were rescued as pBluescript phagemids. Subsequently, positive clones were sequenced bi-directionally and the sequence analysis was performed using LaserGene software (release 3.05:DNASTAR, Madson, WI, USA).

#### 5’ and 3’ Rapid Amplification of cDNA Ends (RACE)

To clone CREB’s from catfish ovary, 5’ and 3’ RACE, as described earlier [13], was carried out using the gene specific primers as listed in Table 1.

### Northern blot analysis

About 5 μg of poly (A)^+^ RNA was obtained from ovarian follicles of different stages and mature testis as described earlier [13]. The RNA was resolved on 1.2% denaturing formaldehyde-agarose gel and capillary transferred onto nylon membranes. cDNA fragments for single partial cDNA CREB (common for all forms of CREB), as well as catfish CREB were radiolabelled and used as probes separately for each blot. The blots were stripped and re-hybridized with *β-actin* probe that served as internal control. Signals were detected using a Fuji BSA2000 Phosphorimager. The signals were quantified by densitometry using NIH image J software.

### Semi-quantitative RT-PCR

A semi-quantitative RT-PCR analysis as described by Kwon *et al*.[16] was employed to study the tissue distribution pattern and expression of CREBs’ during natural ovarian cycle in tilapia. Gene specific primers used in PCR reactions were shown in Table 1. qPCR was not performed as the variations among all the three CREBs are mostly in UTR regions.

### qRT-PCR

Expression of CREB in catfish during different stages of reproductive cycle and hCG-induced oocyte maturation were analyzed by quantitative RT-PCR as described earlier [12].Total RNA was isolated from different stages of ovary as well as during hCG-induction, *in vivo* and *in vitro*. First strand cDNA was synthesized using random hexamers and Superscript III cDNA synthesis kit (Invitrogen) with 5 μg of total RNA. The cDNA template was used in qPCR reaction with *CREB* and *β-actin* primers (**Table 1**) and amplifications and fluorescence detection were performed on ABIPrism 7500 (Applied Biosystems) real-time PCR machine under the manufacturer’s universal thermal cycling conditions. Cycle threshold (CT) values were recorded during exponential phase of PCR amplification, the expression of *CREB* was normalized to that of *β-actin* (ΔCT = *CREB* CT - *β-actin* CT) and abundance of *CREB* mRNA was calculated using the formula 2^-ΔΔCT^. All the RT-PCR data was presented as mean ± SEM and statistical analysis was performed using Graphpad Prism software. Differences between groups were analyzed by ANOVA following Kruskal-Wallis’ test and *P* values ≤0.05 were considered significant.

## Results

### Cloning and sequence analysis of CREBs

A partial cDNA of 405 nt was obtained from the ovarian follicles using a set of degenerate primers by RT-PCR. The identity of the cloned cDNA was confirmed by BLAST analysis. This partial CREB cDNA was used as a probe for the extensive screening of tilapia ovarian and testicular cDNA libraries. After three rounds of cDNA library screening, several positive clones were identified and sequenced. Sequence analysis revealed multiple forms of CREBs. CREB1 cDNA, isolated from testis cDNA library was 1278 nt long with an ORF of 990 nt encoding for a protein of 330 amino acids. CREB2, cloned from tilapia ovary cDNA library was 2826 nt in length with an ORF of 954 nt encoding for a protein of 318 amino acids. Though they differ in few amino acids at N-terminal, they both have the characteristic kinase inducible and DNA binding zinc finger domains. However, CREB2 has a very long 3' UTR (1753 nt) relative to CREB1. In addition to CREB2, in the ovary a 3’UTR variant form was identified and designated as CREB3. CREB3 was 2757 nt in length with an ORF same as CREB2, but has few nucleotides shorter than 3' UTR of CREB2 (1684nt).

**Fig 1:**
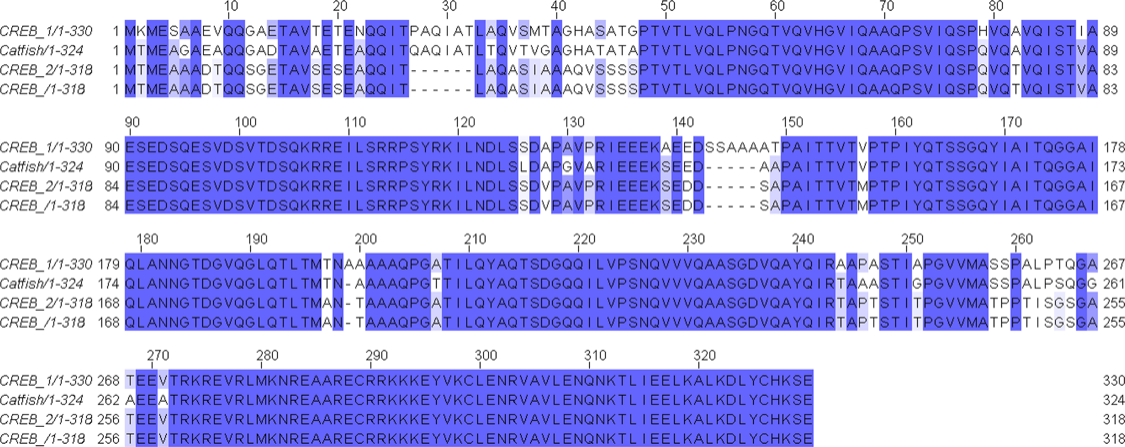
Clustal W multiple alignment of tilapia and catfish CREB deduced amino acid sequences.

**Fig 2:**
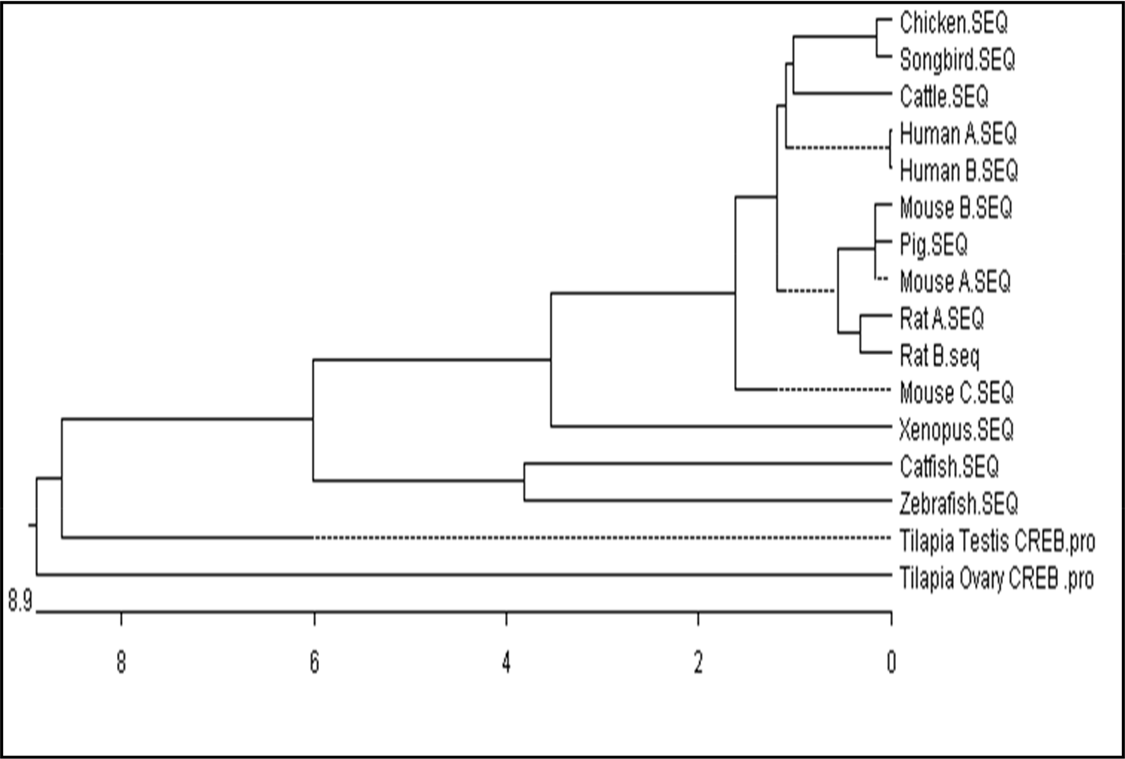
Cladogram showing phylogenetic analysis of the Nile tilapia and catfish CREBs with other vertebrate analogs. (Accession no.: Human A NM_004379; Human B NM_134442; Mouse C NM_001037726; *Xenopus* NM_001086603;Mouse A NM_009952; Mouse B NM_133828; Rat A NM_ 134443; Rat B NM_031017; Chicken NM_ 204450; Zebrafish NM_ 200909; Pig NM_001099929; Cattle NM_174285; Songbird NM_001048256).

In order to extend our studies in catfish, we intended to study the expression pattern of CREBs in catfish. Using the same set of degenerate primers, a partial cDNA of 405 nt that was quite similar to tilapia CREB1 was obtained from the ovarian follicles of catfish. This partial cDNA was used to isolate and identify CREBs in catfish. However, using 5' and 3' RACE strategies, a single CREB that is similar to tilapia CREB1 was isolated. The catfish CREB was 1398 nt with an ORF of 975 bp encoding a protein of 324 amino acids. All CREB nucleotide sequences are submitted to GenBank. An amino acid sequence alignment and a phylogenic tree are shown in **Figs 1 and 2**. The putative proteins from both tilapia and catfish are highly conserved encompassing signature domains such as kinase inducible and zinc finger regions.

### Tissue expression pattern of CREBs

**Fig 3:**
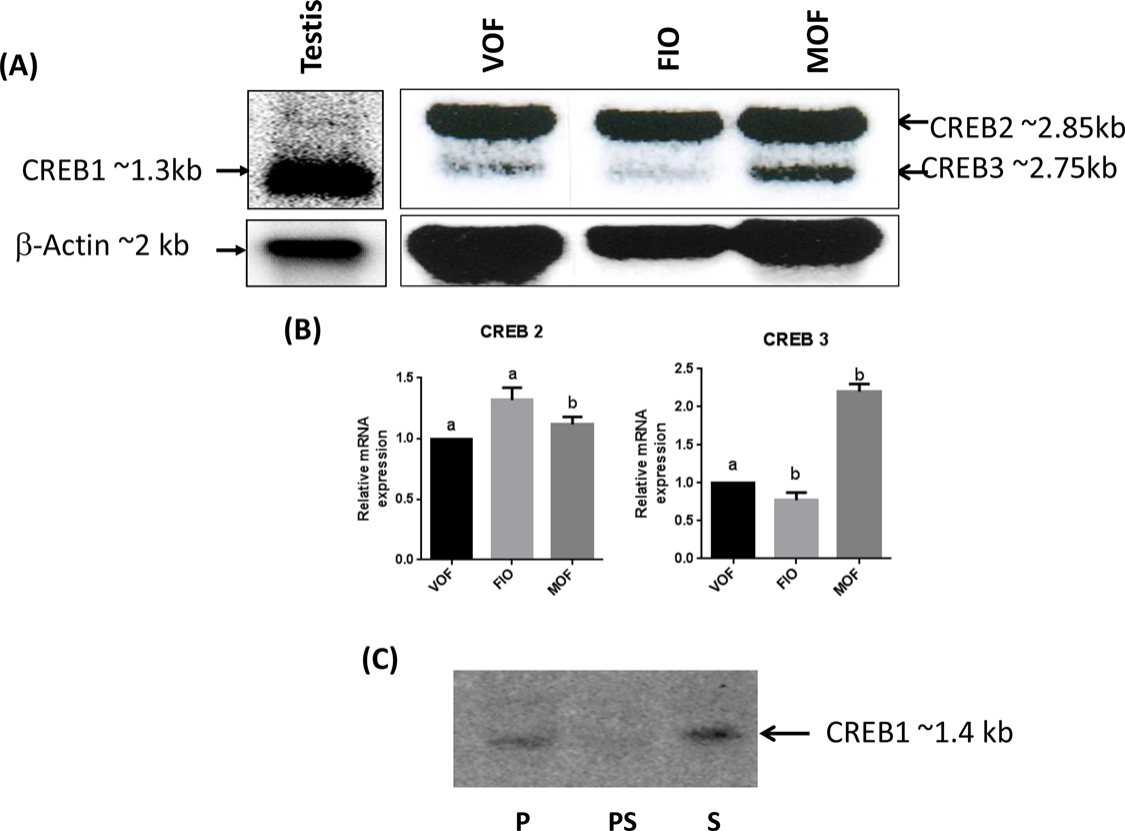
Northern blot analysis of CREBs. **(A)** Expression of CREBs in tilapia ovary and testis **(**VOF; Vitellogenic Ovarian Follicle; FIO, Full grown Immature Ovarian Follicle; MOF, Mature Ovarian follicle). **(B)** Representative densitometric analysis of CREB2 and CREB3 of tilapia. Bars depicting same letter are significantly different from each other (mean ±SEM of two independent experiments). **(C)** Northern blot analysis of CREBs catfish ovary **(**P, Preparatory; PS, Pre-spawning; S, Spawning). *Note*: only one form of CREB (~ 1.3 kb) in tilapia testis [CREB 1] while two forms of CREB (~2.85 [CREB 2] and ~2.75 kb [CREB 3]) in tilapia ovary.

In order to detect the different CREB mRNAs cloned from Nile tilapia, Northern blot analysis was performed using a partial CREB cDNA fragment. Northern blot analysis detected a single transcript of ~1.3 kb (CREB1) in testis, whereas in ovary, two transcripts of ~2.75 (CREB 3) and ~2.85 (CREB 2) kb were detected thus confirming our cloning analysis (**Fig 3A and 3B**). Further, CREB2 showed a very strong signal in ovarian follicles in three different stages with a slight increase in full-grown immature follicles while CREB3 signal was relatively faint but expression was abundant during spawning phase (day) (**Fig 3A and 3B**). Owing to high sequence similarity, the probe in Northern blot could detect CREB1 only in testis, but not in ovarian follicles. This high degree of sequence similarity also posed a limitation to design real-time quantitative PCR assay. Therefore, we had employed semi-quantitative RT-PCR to analyze the expression of Nile tilapia CREBs in different tissues as well as during oocyte maturation.

**Fig 4:**
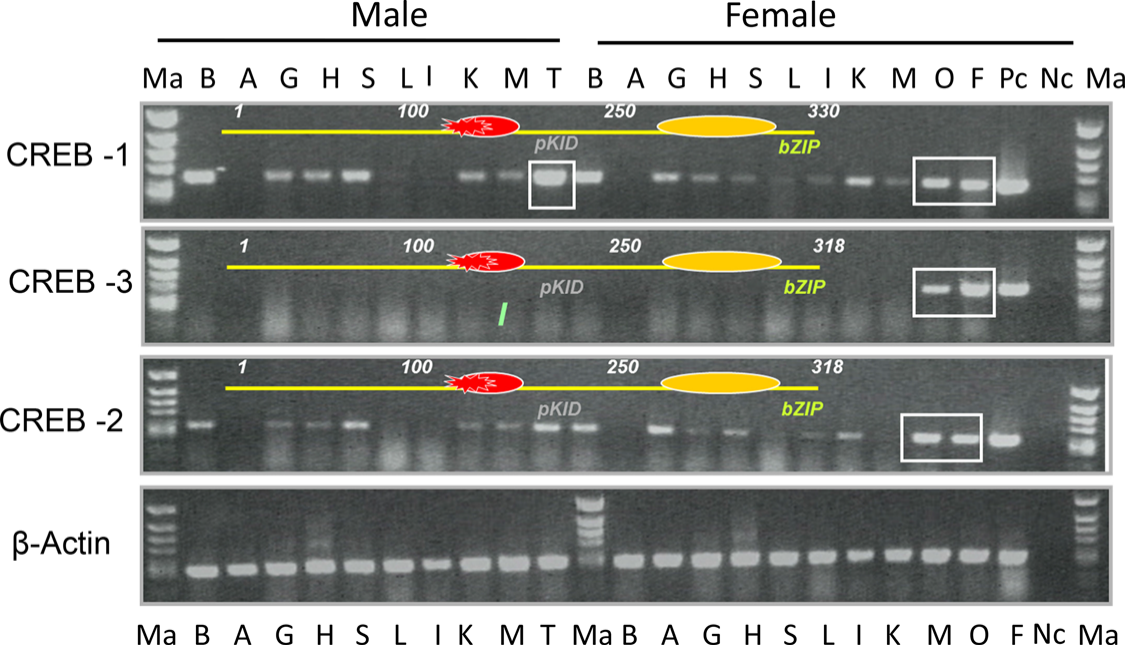
Tissue distribution pattern of tilapia CREBs as determined by semi-quantitative RT-PCR. A plasmid DNA of CREB partial cDNA was used as positive control (PC) and PCR reaction without RT was used as negative control (NC). (Ma, Marker; B, Brain; A, Adrenal; H, Heart; S, Spleen; L, Lung; I, Intestine; K, Kidney; M, Muscle; T, Testis; O, Ovary; F, Ovarian follicle).

Semi-quantitative RT-PCR analysis showed that CREB1 and CREB2 transcripts were detected in most of the tissues analyzed and the expression was predominant in brain, gonads, kidney and spleen (**Fig 4**). There was no appreciable difference in CREB1 and CREB2 expression pattern between male and female tilapia. Interestingly, expression of CREB3 was exclusively found in ovary and ovarian follicles, while it was undetectable in all other tissues analyzed in both sexes (**Fig 4**).

In contrast to multiple forms of CREBs in the Nile tilapia, only a single transcript of CREB was detected in catfish ovary by Northern blot analysis (**Fig 3C**).Tissue expression pattern of catfish CREB was similar to tilapia CREB1 (data not shown).

### Expression of tilapia CREBs in natural ovarian cycle and hCG-induced maturation

**Fig 5:**
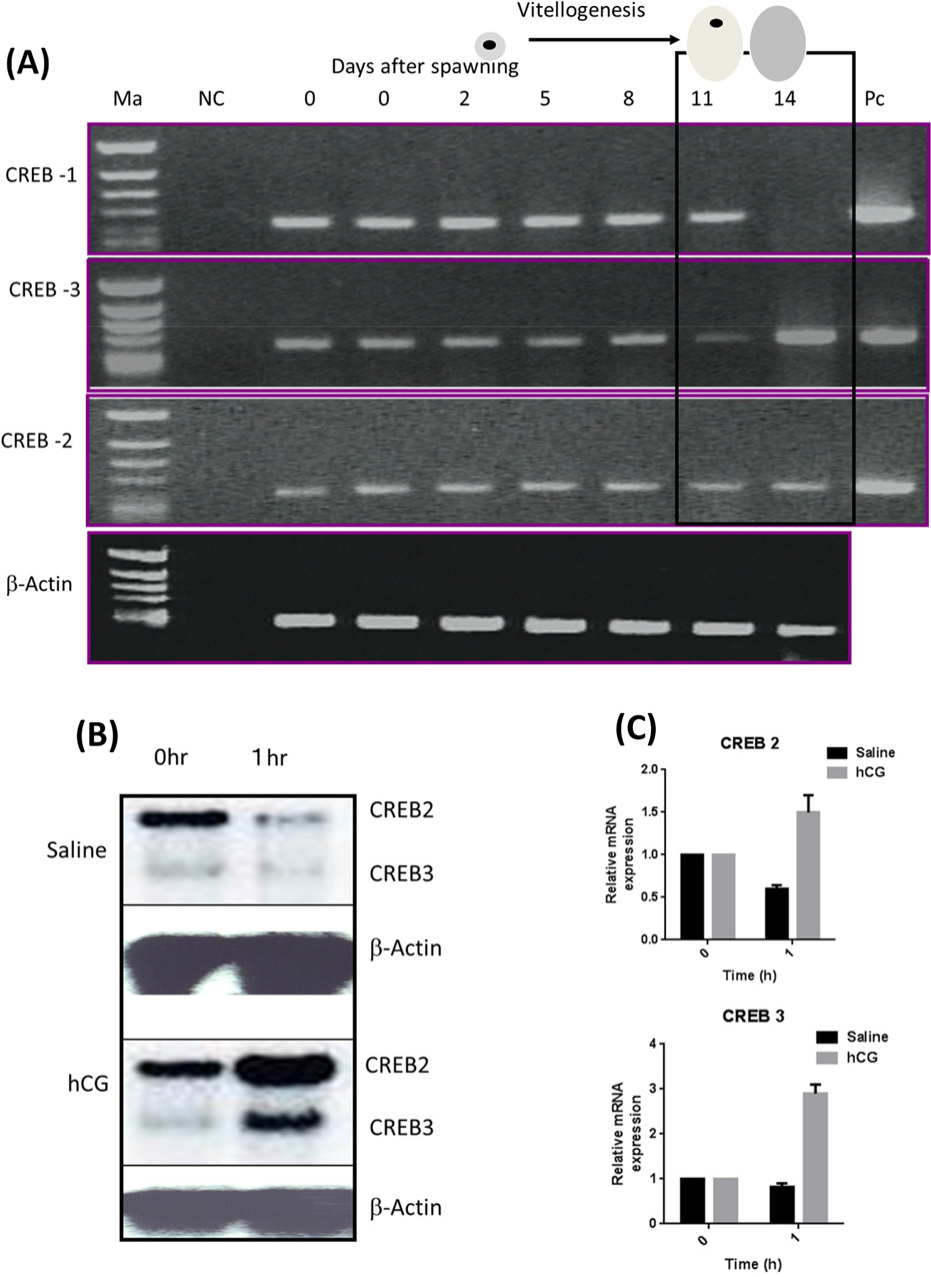
Expression of tilapia CREBs during: (A) natural ovarian cycle and (B) hCG induced -oocyte maturation as determined by RT-PCR and Northern Blot respectively. A plasmid DNA with CREB partial cDNA was used as positive control (PC) and PCR reaction without RT was used as negative control (NC) (Ma, Marker). (C) Densitometric analysis of CREB2 and CREB3 (mean ±SEM of two independent experiments).

During natural ovarian cycle of the Nile tilapia, CREB1 expression remained stable throughout vitellogenesis and expression was down-regulated on the day of spawning. In contrast, CREB2 expression was unchanged throughout the cycle with a minor elevation on day 14. Intriguingly, CREB3 expression was found to be the same from 0 to 8 days after spawning, then decreased in full-grown immature follicles (day 11) and a dramatic increase on day 14 (spawning) was noticed. Taken together these results shows, diminished expression of CREB1 and a great augment in expression of CREB3 and a minor elevation of CREB 2 during spawning were observed in natural ovarian cycle of tilapia (**Fig 5A**), indirectly support their role in oocyte growth and meiotic maturation.

Further we sought to determine the expression of CREB2 and CREB3 following hCG- induced oocyte maturation. Northern blot analysis demonstrated that expression of CREB2 increased about 1.5 folds 1 hour after hCG-injection. CREB3 expression before hCG-injection was very low and its expression induced 3 folds 1 hour after hCG-injection (**Fig 5B and 5C**). As a control, tilapias were also injected with physiological saline, which showed slight decrease in CREB2 and CREB3 expression (**Fig 5B and 5C**). These results strongly support the expression pattern of CREB2 and CREB3 during natural ovarian cycle and confirmed the overexpression of CREB3 and to some extent CREB2 during spawning (on day 14) in the Nile tilapia.

### Expression of catfish CREB in natural ovarian cycle and hCG-induced maturation

**Fig 6:**
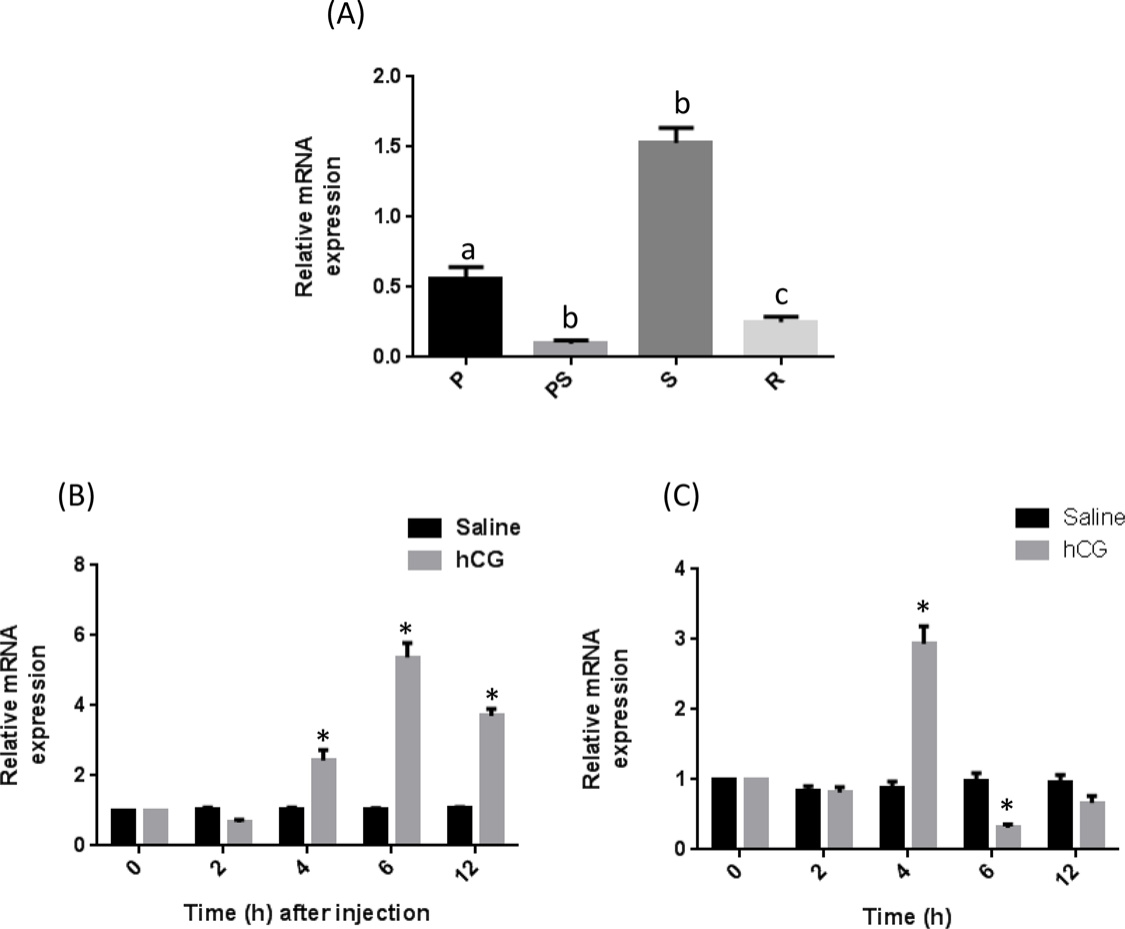
(A) Expression of catfish CREB during natural ovarian cycle (A) and hCG induced oocyte maturation *in vivo* (B) and *in vitro* (C) as determined by real-time RT-PCR. Data are mean±SEM of two independent experiments (n = 3). Bars depicting same letter are significantly different from each other and bars depicting * are statistically significant compared to 0 hours. A plasmid DNA with CREB partial cDNA was used as positive control and PCR reaction without RT was used as negative control.

In catfish reproductive cycle, CREB expression was low in pre-spawning and regressed/resting phases and a maximum expression was noticed during spawning phase (**Fig 6A**). In catfish, both *in vivo* and *in vitro* hCG-induced oocyte maturation, CREB mRNA levels augmented rapidly (**Fig 6B and 6C**).

## Discussion

cAMP mediates communication between cells in many biological processes such as cellular homeostasis, cell proliferation and death, neuronal plasticity, long term memory, steroid hormone and glucose metabolism etc., [17]. cAMP employs the transcription factor CREB which is activated by phosphorylation and modulates gene expression by binding to cAMP responsive elements on gene promoters. cAMP has multiple roles in fish oocytes. For example, cAMP is known to be critical for the synthesis of both E_2_ and 17α, 20β-DP. Prophase I arrest maintenance requires a state of decreased intra-oocyte cAMP. On the other hand, gonadotropins, in large part, mediate their actions on several steroidogenic enzyme genes through cAMP [18–20]. Together, the identification of cAMP response elements on both *20β-HSD* and *Cyp19a1a* promoters added an enigma to our understanding of molecular mechanism of shift in steroidogenesis in fish oocytes [1,5,6]. We speculated that CREBs’ might temporally regulate the different stages of oocyte maturation [5,6]. Surprisingly, we identified multiple forms of CREB in tilapia and a single form of CREB in catfish.

CREB1 and CREB2 are ubiquitously expressed in several tissues in addition to ovary and testis. Stable expression of CREB2 throughout of tilapia ovarian cycle with an elevation during spawning indicates its possible role in maintaining basal level of steroidogenic enzymes in particular *20β-HSD* and to some extent *Cyp19a1a*. This notion is documented well by the observation of CREB2 overexpression following hCG-induction. In mammals, CREB and SF-1 bind to *Cyp19a1a* gene in co-operative manner to mediate cAMP action in granulosa cells [21–23]. Although, putative cAMP responsive elements were identified on fish *Cyp19a1a* promoters, either a direct role or interactions with other factors remain elusive. However, CREB1 expression pattern correlates well with *Cyp19a1a* expression and possibly involve in down regulation of *Cyp19a1a* expression during steroidogenic shift [7,8]. CREB3 seems to play a very prominent role in the up regulation of *20β-HSD* expression vis-à-vis final oocyte maturation. This contention is supported by i) the exclusive presence of CREB3 in ovary and ovarian follicles, ii) synergistic expression pattern of CREB3 with *20β-HSD* in natural ovarian cycle, iii) over expression of CREB3 preceding *20β-HSD* during hCG-induced oocyte maturation. Therefore, these results strongly support that shift in steroidogenesis at transcriptional level is possibly regulated by multiple forms of CREBs in tilapia. It seems CREB1 regulates *Cyp19a1a* while CREB3 as well as CREB2 regulates *20β-HSD* expression. However, we could not be able to provide the direct evidence to this notion, due to the lack of fish specific CREBs recombinants and future studies shall address this fact. Nevertheless, using natural and hCG-induced oocyte maturation with two fish different models and identification of CRE motif [15] (in fish *20β-HSD* promoter supports this concept strongly.

To extend these observations in an annual breeder, catfish, similar studies were conducted. As opposed to tilapia, a single form of CREB that is homologous to tilapia CREB1/2 was identified. In spite of repeated attempts we could not able to get the variant form of CREB in ovary. Unlike tilapia, we did not use testicular tissue for cloning CREB. Expression pattern of CREB in catfish correlate more or less with *20β-HSD* expression and enzyme activity in natural and hCG-induced oocyte maturation. Thus, we presume that CREB might regulate both *Cyp19a1a* and *20β-HSD* as the two events of ovarian cycle, vitellogenesis and maturation are widely spaced in catfish as opposed to the short time in tilapia. Another explanation could be the interaction of multiple transcription factors to modulate expression of these two enzymes.

In conclusion, multiple forms of CREBs were identified in tilapia ovary for the first time in any lower vertebrates. Based on their expression patterns, CREB1 could be involved in regulating *Cyp19a1a* and an alternatively spliced (UTR variant) CREB3 is exclusively expressed in ovary playing a major role in shift in steroidogenesis by targeting *20β-HSD*. Further, hCG-induced over expression of CREB3 as well as CREB2 demonstrate the robust action of these correlates to promote final oocyte maturation by transcriptionally regulating [15] *20β-HSD* expression and function [6]. In catfish, a single form of CREB1 was identified that may probably implicated in final oocyte maturation and to some extent in vitellogenesis.
